# Recurrent massive hemoptysis from distal pulmonary pseudoaneurysms complicating invasive aspergillosis in a teenager

**DOI:** 10.1016/j.radcr.2022.07.095

**Published:** 2022-08-11

**Authors:** Caroline Mora-Soize, Aline Carsin-Vu, Gratiela Mac Caby, Nasredine Belkessa, Claude Marcus, Sebastien Soize

**Affiliations:** a Department of Pediatric Radiology, American Memorial Hospital, CHU de Reims, Université de Reims Champagne-Ardenne, 47 rue Cognacq-Jay, 51092, Reims, France; b Department of Diagnostic and Interventional Radiology, Hôpital Robert Debré, CHU de Reims, 51 avenue du Général Koening, Université de Reims Champagne-Ardenne, 51092, Reims, France; c Department of Radiology, Hôpital Maison Blanche, CHU de Reims, Université de Reims Champagne-Ardenne, 45 rue Cognacq-Jay, 51092, Reims, France

**Keywords:** Aspergillosis, Hemoptysis, Pulmonary, Aneurysm, Case report, Children, CT, computed tomography, CTA, computed tomography angiography, CR, chest radiography

## Abstract

Invasive pulmonary aspergillosis in children rarely complicates life-threatening massive hemoptysis. Here, we report the case of a 15-year-old girl with acute lymphoblastic leukemia who was hospitalized for fever and medullary aplasia 1 month after beginning chemotherapy for invasive pulmonary aspergillosis. Despite voriconazole and caspofungine treatment, excavation of some lesions caused a unilateral small pneumothorax and bilateral pleural effusion, justifying intensive care management. The massive hemoptysis that occurred on day 23 was complicated with heart failure, and the patient was promptly resuscitated. Fibroscopy and computed tomography angiography (CTA) did not reveal the origin or cause of the bleeding. A second massive bleeding event occurred on day 32, and heart failure resolved after 10min of low flow. A new CTA showed 2 pseudoaneurysms of the subsegmental pulmonary arteries that were treated with embolization. Sedation was gradually decreased owing to improvement in respiratory status, but the patient did not regain consciousness because of deep brain sequelae. A limitation of care was decided upon, and the patient died in the following weeks. Massive hemoptysis is a rare life-threatening complication of invasive pulmonary aspergillosis, especially in children. Pulmonary artery pseudoaneurysms are unusual and should be detected as soon as possible to guide therapy. Intensive care management should be followed by embolization if the patient is stable; otherwise, surgery is indicated, ideally after identifying the source of bleeding by CTA or bronchoscopy. Early CTA follow-up can be proposed if the source of bleeding is still unknown as pseudoaneurysms can appear or grow rapidly.

## Background

Invasive pulmonary aspergillosis is one of the most frequent opportunistic invasive fungal infections occurring in a background of severe immune depression [[1], [2], [3], [4]–5]. The majority of cases occur in patients with malignant hematological diseases, particularly during chemotherapy induction or consolidation phases [1–5]. The principal risk factors are profound and prolonged neutropenia, perturbed phagocyte function, and cellular immune deficiency [4,5]. Despite improvements in its diagnosis, prevention, and treatment, invasive aspergillosis is a major cause of morbimortality in immunocompromised patients, notably children, and still responsible for a high mortality rate [1–6]. Its frequency tends to increase with improvement in the care of the causal disease; however, treatment success and long-term survival after invasive aspergillosis diagnosis remain suboptimal [5]. The diagnosis can be difficult because of the lack of specificity of symptoms or isolated fever; clinically, it often presents as nonspecific pneumonia not responding to conventional antibiotherapy. Empirical antifungal treatment is often used in neutropenic children with fever who are not improving despite treatment with antibiotics; this one is then adapted to infectious samples analysis [1–6]. Small to mild hemoptysis is sometimes encountered in patients with invasive aspergillosis, whereas massive hemoptysis with blood loss >240 mL or >8 mL/kg in 24 hours is a much rarer but frequently fatal complication (5-10% of invasive aspergillosis patients) [[6], [7], [8]–9]. Bleeding commonly originates from bronchial arteries (>50%), whereas other locations such as intercostal arteries (20%), internal thoracic arteries (15%), inferior phrenic arteries (7%), and pulmonary arterial branches (4%) are more rarely encountered [7]. To our knowledge, only a few cases of massive hemoptysis originating from pulmonary arteries in adults with invasive aspergillosis has been described in the literature and has never been reported in the pediatric population [[10], [11], [12], [13], [14], [15], [16], [17]–18].

## Case report

**Fig. 1:**
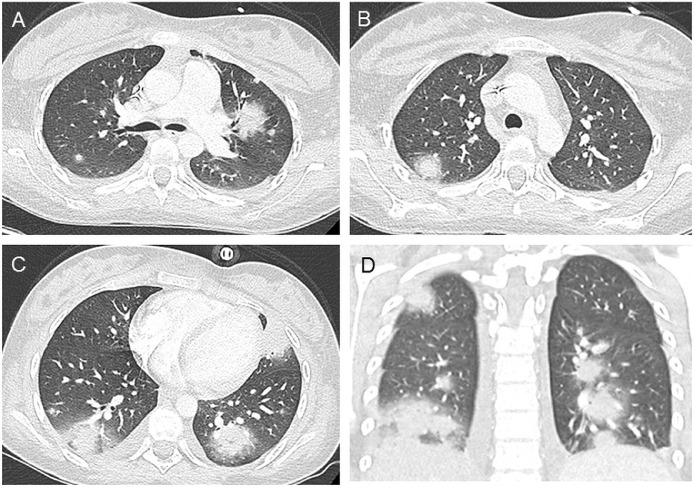
Baseline contrast-enhanced chest computed tomography (CT). Baseline chest CT was performed for persistent febrile medullary aplasia despite the administration of piperacillin/tazobactam and amikacin, followed by ciprofloxacin and linezolide. Axial (A, B, C) and coronal (D) slices show bilateral nodular opacities of mixed topography (central and subpleural) surrounded by halos of ground-glass opacities. Some lesions began to be excavated. These patterns were highly suggestive of invasive pulmonary aspergillosis.

**Fig. 2:**
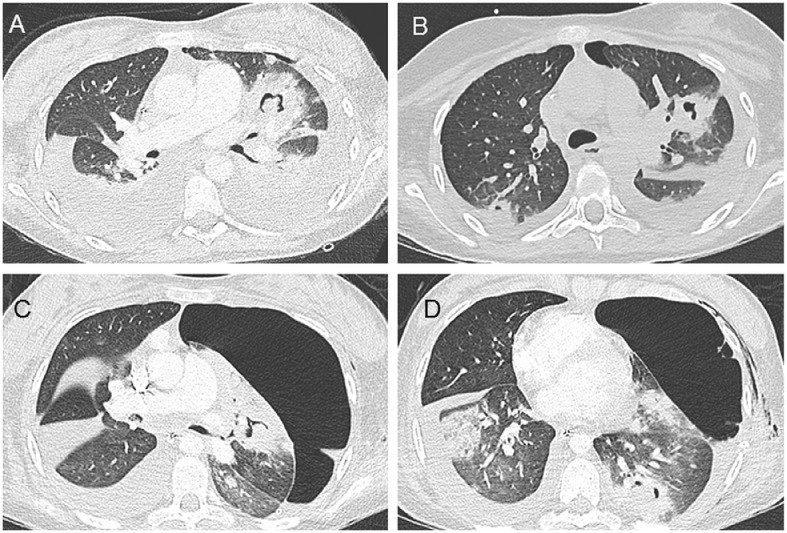
Second and third contrast-enhanced chest computed tomography (CT). Thirteen days after the baseline chest CT, a second CT (A, B) was performed because of worsening respiratory status and a small left pneumothorax, bilateral pleural effusion of mild abundance, and obvious cavitation of preexisting lesions. Thirty days after the baseline chest CT, a third CT (C, D) was performed after the first episode of massive hemoptysis complicated by heart failure with successful resuscitation. Intra-alveolar and endobronchial hemorrhage with a marked increase in pneumothorax were observed. The aspergillosis lesions (nodes and cavities) remained stable.

The present report follows the Consensus-based Clinical Case Reporting (CARE) Guidelines [19,20]. A 15-year-old girl with high-risk acute lymphoblastic leukemia treated with asparaginase was hospitalized because of fever and medullary aplasia 1 month after beginning chemotherapy. She was administered piperacillin/tazobactam plus amikacin with the rapid addition of ciprofloxacin and linezolide. Because of persistent inflammatory syndrome on day 2 after admission, she underwent thoracoabdominal contrast-enhanced computed tomography (CT), which showed pulmonary lesions compatible with invasive pulmonary aspergillosis (Fig. 1). Tests for specific antigens were positive and treatment with voriconazole and caspofungine was initiated in association with oxygen therapy. Fifteen days after admission, despite cytologic remission, her respiratory status worsened, prompting another chest CT scan that revealed a unilateral low-abundance pneumothorax, bilateral pleural effusion, and excavation of preexisting lesions (Fig. 2). These findings justified her transfer to intensive care for pleural drain installation and adaptation to anti-infectious treatment (faced with suspicion of pulmonary superinfection).

**Fig. 3:**
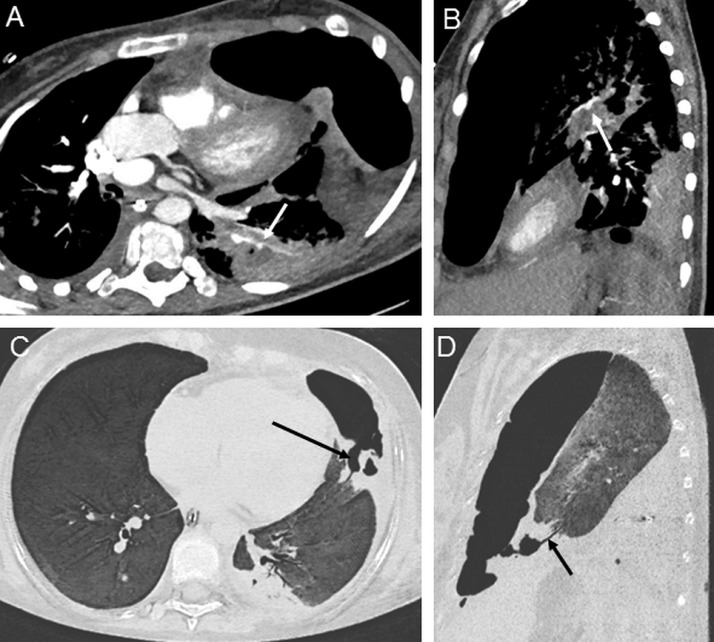
Pulmonary computed tomography angiography (CTA). Thirty days after the baseline chest CT, a second episode of massive hemoptysis occurred that was complicated by heart failure with successful resuscitation. Pulmonary CTA was performed and showed 2 pseudoaneurysms that developed from the subsegmental arteries of the left lower lobe (A) and the anterior segment of the culmen (B) without active bleeding. The invasive aspergillosis lesions were stable, except for the appearance of ground-glass micronodes due to intra-alveolar hemorrhage. A bronchopleural breach (C, D) was visible, which could explain the persistence of pneumothorax despite drainage.

The first episode of massive hemoptysis complicated by heart failure occurred on day 23 after admission, justifying intubation, blood transfusions, and the use of vasopressor amines. The enhanced chest CT scan performed after stabilization showed intra-alveolar and endobronchial hemorrhage with a marked increase in the pneumothorax (Fig. 2). Fibroscopy revealed diffuse inflammatory mucosa with bloody secretions without active bleeding. A second episode of massive hemoptysis (2-3 L) with heart failure occurred on day 32, with 10 min of low flow. Once the patient was stable, a new enhanced chest CT scan revealed 2 pseudoaneurysms that developed from the subsegmental pulmonary arteries (Fig. 3) of the left lower lobe and anterior segment of the culmen, without active bleeding. The invasive aspergillosis lesions were stable, except for the appearance of ground-glass micronodes due to intra-alveolar hemorrhage. A bronchopleural breach (Fig. 3) was clearly visible, which explains the persistence of the pneumothorax despite drainage.

**Fig. 4:**
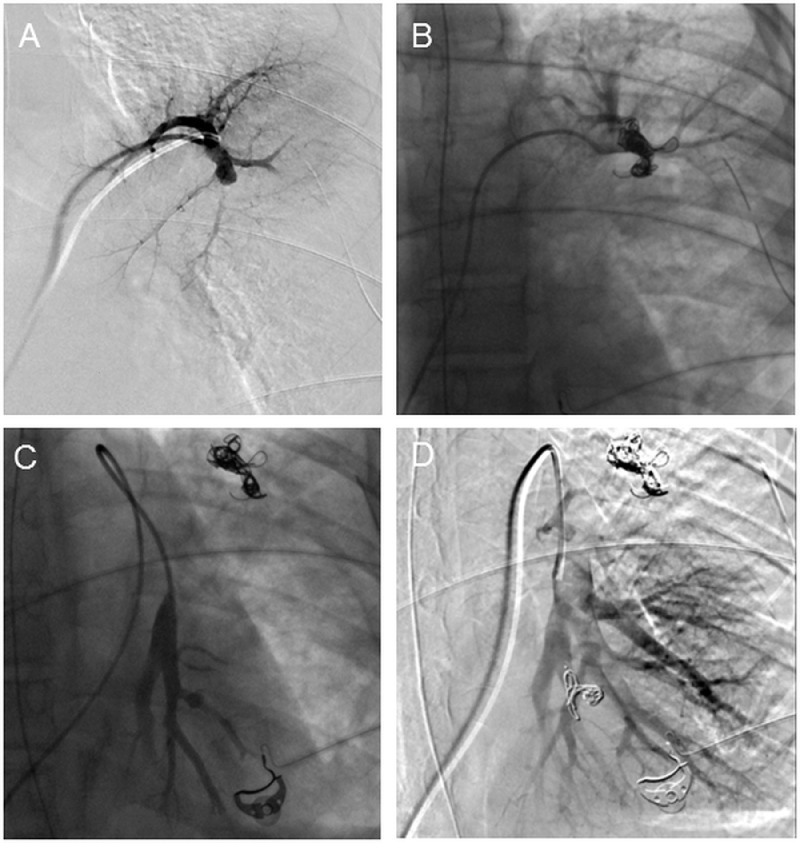
Selective embolization. Digital subtraction angiography confirmed 2 pseudoaneurysms of subsegmental pulmonary artery branches (A: artery from the anterior segment of the culmen, B: postero-basal artery of the left inferior lobe) that were successfully occluded with coils (C, D).

On the same day, selective embolization of the pulmonary artery branches (Fig. 4) allowed occlusion of the pseudoaneurysms, limiting the risk of recurrent bleeding, and a protective tracheostomy tube was placed considering the risk of clot migration to help with ventilatory weaning. The respiratory state improved with gradual achievement of ambient air ventilation on day 42, without recurrence of respiratory distress or bleeding. Chest CT scans were satisfactory, with no new aneurysm, lower lobe atelectasis due to embolization, or persistence of the bronchopleural breach. There was no indication for surgery for this breach (lobectomy or pulmonary plug) considering the absence of hemoptysis recurrence and the risk/benefit ratio.

Sedation was gradually decreased owing to the improvement in respiratory status; however, the patient did not regain consciousness. An electroencephalogram was nonreactive and showed low activity. Encephalic magnetic resonance imaging showed anoxo-ischemic lesions after heart failure in the central gray nuclei with diffuse cortico-subcortical atrophy. A limitation of care was decided in a collegial meeting given the poor neurological prognosis, and the patient died in the following weeks.

## Discussion

This case highlights the poor prognosis of children with invasive aspergillosis complicated by massive hemoptysis despite appropriate treatment and the need for immediate angiography and repeated computed tomography angiography (CTA) when the source of bleeding cannot be detected because of the high risk of life-threatening bleeding recurrence.

In children with invasive aspergillosis, massive hemoptysis secondary to vascular breaches and/or arterial pseudoaneurysms is rare. These vascular lesions usually appear during the bone marrow regeneration phase, when neutrophil migration to the affected regions by *Aspergillus* generates an inflammatory local reaction causing an increase in vascular permeability and promotes invasion of blood vessels by *Aspergillus*, causing vascular wall damage [21]. In 90% of massive hemoptysis cases, high-pressure bronchial arteries are the source of bleeding; they become dilated and tortuous due to chronic infectious or inflammatory lung diseases, and thus the wall thickening increases progressively with the recruitment of anastomoses and release of angiogenic growth factor leading to vascular breaches and/or arterial pseudoaneurysm creation [21,22]. Similar to the present case, when the bronchial arteries are not the source of bleeding, other sources must be sought, including non-bronchial systemic arteries or collaterals that can be recruited secondary to chronic lung inflammation in 5% of cases and pulmonary arteries in 5% of cases (eg, rupture of a Rasmussen's aneurysm) [[22], [23]–24].

Chest radiography (CR), CTA, and bronchoscopy are the most frequently used modalities to localize the bleeding site and are used alone or in combination, depending on institutional practice, availability, and patient stability [25,26].

Studies comparing diagnostic methods for detecting the site and cause of bleeding in life-threatening hemoptysis are available for adults but not for the pediatric population [[27], [28]–29]. These studies suggest that CTA is superior to CR and bronchoscopy for detecting the site and cause of bleeding. Indeed, CTA localizes the site of bleeding in 70-88.5% compared to 73% for bronchoscopy and 46% for CR, and CTA determined the cause of bleeding in 77% of cases compared to 8% for bronchoscopy and 35% for CR [27–29]. In a study of 40 patients presenting with hemoptysis who had a normal bronchoscopy, a subsequent CT scan detected an etiology of hemoptysis in 50% of the patients [29]. Thus, CTA is the preferred modality in patients who have adequate oxygenation, ventilation, and hemodynamic stability. Typical imaging patterns included focal outpouchings of contrast adjacent to a branch of the artery following the same contrast density as the pulmonary artery in all phases of the study. Flexible bronchoscopy is useful when the patient is too unstable to undergo diagnostic imaging studies or when CT imaging cannot localize the bleeding site [22,25,26].

In addition to these modalities, angiography can be used when the site or cause of bleeding is still unknown and to provide endovascular treatment [26,30]. Angiographic localization of the bleeding site can be technically challenging and time-consuming and requires a significant contrast load; therefore, pre-procedural chest CT and/or bronchoscopy to help localize the bleeding site is valuable [24,26,30]. A study showed that CTA was superior to angiography for detecting bleeding from the bronchial and non-bronchial systemic arteries [30].

There is no recommendation for the diagnostic management of massive hemoptysis in invasive aspergillosis patients without a clearly identified origin after CTA and bronchoscopy. The case reported here highlights the need for repeated investigations as the risk of rebleeding is very high and pseudoaneurysms can grow and rapidly become visible. In case a pseudoaneurysm is discovered, aneurysm preventive treatment as quickly as possible is indicated as aneurysms can modify the patient's prognosis. The recommended approach for mycotic aneurysms is prolonged antimicrobial therapy. There are no guidelines on how to best manage life-threatening hemoptysis. Initial management should focus on airway control, volume resuscitation (if needed), and correction of any bleeding disorder [22,25] Patients presenting with life-threatening hemoptysis should be managed in an intensive care setting. Intubation for airway control is typically required in life-threatening hemoptysis, possibly requiring flexible bronchoscopy [25]. Therapeutic techniques to attempt hemostasis via the bronchoscope include cold saline lavage, local instillation of topical vasoconstrictive agents (epinephrine), and insertion of balloon blockers [22,25].

Angiography is potentially both diagnostic and therapeutic. Following initial stabilization and localization of the bleeding site, embolization is usually the first-line therapy for life-threatening hemoptysis. Once the bleeding vessel is identified, super-selective arterial embolization is performed. If the interventional radiologist is unable to localize the site of bleeding in the bronchial circulation, non-bronchial systemic and pulmonary circulations can be sequentially evaluated for bleeding. Studies suggest that a systematic search for non-bronchial systemic collaterals reduces the recurrence rate and leads to better overall hemoptysis control [22,26,30]. Due to the availability of safe and effective endovascular embolization techniques, embolization has largely replaced emergent surgery for the management of life-threatening hemoptysis.

The specific indications for surgical intervention in the setting of life-threatening hemoptysis include technical failure of the embolization procedure, recurrent hemoptysis despite multiple embolizations, maximum medical therapy, or life-threatening circumstances during the hemoptysis episode that does not permit the safe performance of an interventional radiological procedure. Emergency cardiothoracic surgery in itself has a mortality rate of 40% [30].

In conclusion, massive hemoptysis is a rare life-threatening complication of pulmonary invasive aspergillosis, especially in children. Pulmonary artery pseudoaneurysms are unusual and should be detected as soon as possible to guide therapy. Intensive care management should be followed by embolization if the patient is stable; otherwise, surgery is indicated, ideally after identifying the source of bleeding by CTA or bronchoscopy. Early CTA follow-up can be proposed if the source of bleeding is still unknown as pseudoaneurysms appear or grow rapidly.

## Patient consent

Written informed consent for the publication of this case report was obtained from the patient's parents.
